# The Modulating Role of Sex and Anabolic-Androgenic Steroid Hormones in Cannabinoid Sensitivity

**DOI:** 10.3389/fnbeh.2018.00249

**Published:** 2018-10-26

**Authors:** Dicky Struik, Fabrizio Sanna, Liana Fattore

**Affiliations:** ^1^Department of Biomedical Sciences, University of Cagliari – Cittadella Universitaria di Monserrato, Monserrato, Italy; ^2^CNR Institute of Neuroscience-Cagliari, National Research Council, Rome, Italy

**Keywords:** gonadal hormones, anabolic-androgenic steroids, cannabinoids, dependence, sex, dopamine

## Abstract

Cannabis is the most commonly used illicit drug worldwide. Although its use is associated with multiple adverse health effects, including the risk of developing addiction, recreational and medical cannabis use is being increasing legalized. In addition, use of synthetic cannabinoid drugs is gaining considerable popularity and is associated with mass poisonings and occasional deaths. Delineating factors involved in cannabis use and addiction therefore becomes increasingly important. Similarly to other drugs of abuse, the prevalence of cannabis use and addiction differs remarkably between males and females, suggesting that sex plays a role in regulating cannabinoid sensitivity. Although it remains unclear how sex may affect the initiation and maintenance of cannabis use in humans, animal studies strongly suggest that endogenous sex hormones modulate cannabinoid sensitivity. In addition, synthetic anabolic-androgenic steroids alter substance use and further support the importance of sex steroids in controlling drug sensitivity. The recent discovery that pregnenolone, the precursor of all steroid hormones, controls cannabinoid receptor activation corroborates the link between steroid hormones and the endocannabinoid system. This article reviews the literature regarding the influence of endogenous and synthetic steroid hormones on the endocannabinoid system and cannabinoid action.

## Introduction

Drug use causes considerable harm because of premature death and disability as well as other adverse health effects. The United Nations Office on Drugs and Crime estimated that around 0.6% of the world population suffers from substance use disorders (United Nations Office on Drugs and Crime [UNODC], 2017). Although opioids are considered the most harmful drugs for their addiction potential and negative consequences, cannabis use is a much larger problem when it comes to the number of users. Around 183 million “past-year” cannabis users were reported worldwide in 2015, which is 2.6 times higher than the cumulative number of “past-year” worldwide users of opioids, amphetamines and cocaine, making cannabis the most widely used illicit drug at a global level (United Nations Office on Drugs and Crime [UNODC], 2017). Although worldwide cannabis use has remained stable (3.4% in 1998 versus 3.8% in 2015), the absolute number of cannabis users has increased because of the growing world population, especially in Africa and Asia (United Nations Office on Drugs and Crime [UNODC], 2017). Legalization of marijuana for medical and recreational purposes might increase cannabis use even further (Hopfer, 2014). In addition to traditional marijuana use, the use of synthetic cannabinoids (i.e., designer drugs that mimic the physical and psychological effects of delta-9-tetrahydrocannabinol (THC), the primary active constituent in cannabis) is gaining considerable popularity. Since 2008, when the first synthetic cannabinoid (JWH-018) was detected in the market, at least 169 different synthetic cannabinoids have been discovered (Fattore and Fratta, 2011; European Monitoring Centre for Drugs and Drug Addiction [EMCDDA], 2017). The emergence of synthetic cannabinoids is becoming an increasing concern because of their undetermined addiction potential and adverse health effects (Fattore, 2016; Weinstein et al., 2017; De Luca and Fattore, 2018; Zanda and Fattore, 2018).

Acute toxicity of traditional cannabis use is considered low (Nahas, 1972); yet, long-term cannabis use is associated with serious adverse health effects which include lower birth weight of offspring (maternal cannabis smoking), diminished lifetime achievement, development of psychosis, depression or anxiety, symptoms of chronic bronchitis, motor vehicle accidents, and risk of cannabis addiction (Hall and Degenhardt, 2009; Volkow et al., 2014; United Nations Office on Drugs and Crime [UNODC], 2017). Although the existence of cannabis addiction was disputed in the 1990s, current evidence predicts that around 1 in 10 cannabis users will develop cannabis addiction or dependence (Lopez-Quintero et al., 2011), which is currently defined as cannabis use disorder (CUD) in the fifth edition of the Diagnostic and Statistical Manual for Mental Disorders (5th ed.; DSM-5; American Psychiatric and Association, 2013). CUD is characterized by high cannabis intake over longer periods of time, problems with controlling cannabis use, tolerance, withdrawal signs, craving and negative effects on personal, social and occupational activities (DSM-5).

The demand for CUD treatment is increasing dramatically. The European Monitoring Centre for Drugs and Drug Addiction reported a 50% increase in the number of first-time entrants for CUD treatment in 2011 (European Monitoring Centre for Drugs and Drug Addiction [EMCDDA], 2013). The increasing need for CUD treatment is thought to be driven by the increased availability of cannabis products containing higher concentrations of THC or synthetic cannabinoids (Freeman and Winstock, 2015). Regrettably, current CUD treatment protocols show modest effects only (Budney et al., 2007; Weinstein and Gorelick, 2011). Delineating risk factors involved in the initiation and maintenance of cannabis use therefore becomes increasingly important and critical for optimizing evidence-based prevention and treatment protocols.

**FIGURE 1:**
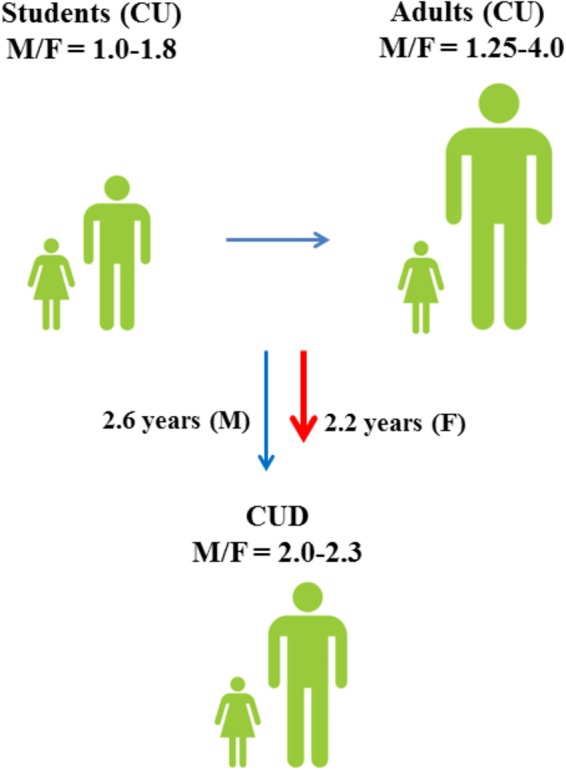
Male to female ratios of lifetime cannabis use (CU) and progression toward cannabis use disorder (CUD) among students (15–16 years) and adults (European Monitoring Centre for Drugs and Drug Addiction [EMCDDA], 2005). Although males have a higher risk of developing CUD (Zhu and Wu, 2017), progression toward CUD is faster in females (Khan et al., 2013).

**FIGURE 2:**
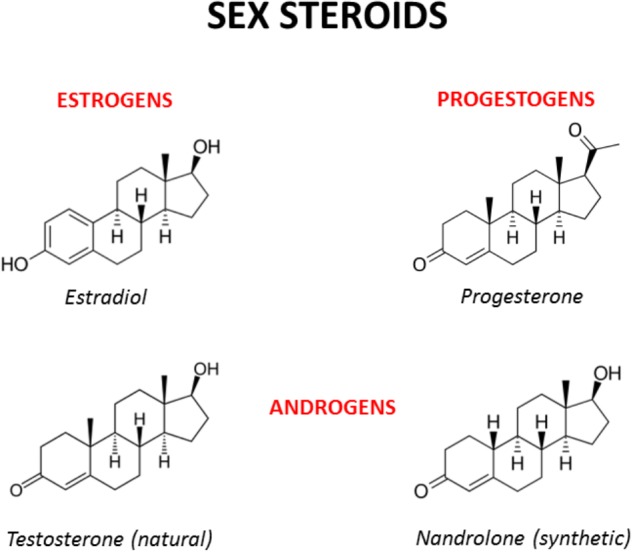
Chemical structures of the main male and female sex hormones and the anabolic-androgenic steroid nandrolone. The strict chemical homology with a common core cyclic structure among natural and synthetic steroids accounts for the relatively high cross-reactivity displayed by sex steroids and AAS at receptor level. The only difference between testosterone and nandrolone (19-nortestosterone) is the methyl group (CH_3_) of nandrolone in position C19 instead of the hydrogen (H) of testosterone, which increases the anabolic activity of nandrolone and is at the basis of its use as a doping drug (see Busardò et al., 2015 and references enclosed).

Similarly to other drugs of abuse, cannabis use differs remarkably between males and females (European Monitoring Centre for Drugs and Drug Addiction [EMCDDA], 2005), indicating a different sensitivity to cannabinoid-induced effects in the two sexes (Davis and Fattore, 2015; Figure 1). Although it remains uncertain which specific biological (i.e., sex) and socio-cultural (i.e., gender) factors affect cannabis use in humans, animal studies strongly suggest the involvement of sex (Fattore and Fratta, 2010) and anabolic-androgenic steroids (AAS) hormones (Struik et al., 2017) as important modulators of cannabinoid sensitivity. This review aims to describe the role of sex differences in cannabis use with reference to the modulating role of sex and AAS hormones (Figure 2) in cannabinoid sensitivity.

## Risk Factors for Cannabis Use

As for other drugs of abuse, both genetic and environmental factors play a role in cannabis use and addiction (Agrawal and Lynskey, 2006; Verweij et al., 2010). Twin studies estimate that the genetic contribution to cannabis use is between 17 and 67%, while the genetic contribution to cannabis addiction is much higher and ranges from 45 to 78% (Verweij et al., 2010; Vink et al., 2010; Distel et al., 2011; Lynskey et al., 2012). Interestingly, the genetic contribution to the initiation of cannabis use increases with age (Distel et al., 2011) and is higher in males than in females (van den Bree et al., 1998). Although it is clear that genetics is an important risk factor in cannabis use and abuse, it has so far proved difficult to identify specific gene variants that alter cannabis sensitivity. At present, most genome-wide association studies (GWAS) failed to detect significant associations between cannabis use and genetic variants (Agrawal et al., 2011; Verweij et al., 2013; Stringer et al., 2016). However, using gene-based testing, four genes that are significantly associated with lifetime cannabis use have been recently identified, which include the neural cell adhesion molecule 1 (NCAM1), the cell adhesion molecule 2 (CADM2), the Short Coiled-Coil Protein (SCOC) and the potassium sodium-activated channel subfamily T member 2 (KCNT2) (Stringer et al., 2016). Interestingly, NCAM1 has been associated with substance abuse (Gelernter et al., 2006) and is part of the NTAD gene cluster (NCAM1-TTC12-ANKK1-DRD2) which is linked to neurogenesis and dopaminergic signaling (Yang et al., 2008). In the most recent GWAS, single-nucleotide polymorphisms (SNPs) in novel antisense transcript RP11-206M11.7, solute carrier family 35 member G1, and the CUB and Sushi multiple domains 1 gene were significantly associated with cannabis dependence (Sherva et al., 2016). However, whether or not these genes contribute to altered cannabinoid action remains unclear. Next to genetic variation, epigenetic-dependent changes in gene expression might contribute to altered cannabinoid sensitivity. Interestingly, a recent study reported increased DNA methylation of the NCAM1 gene in cannabis users compared to control subjects (Gerra et al., 2018).

The vulnerability to initiation of cannabis use and CUD development appears heritable. Yet, numerous social and environmental factors (e.g., age of cannabis use initiation, peer drug use, availability of drugs, low socio-economic status, experience of childhood sexual abuse, cigarette smoking or alcohol drinking during early adolescence) and the presence of pre/comorbid psychopathology (e.g., mood disorders, ADHD, psychosis) are thought to enhance the risk of transitioning from initiation of cannabis use to CUD (reviewed in Courtney et al., 2017). Personality/biological traits, such as impulsivity, schizotypy and sensation-seeking, are also positively correlated with the initiation of cannabis use in adolescents and young adults (Haug et al., 2014; Muro and Rodríguez, 2015).

As for other drugs of abuse, the prevalence of cannabis use differs remarkably between males and females (Figure 1; European Monitoring Centre for Drugs and Drug Addiction [EMCDDA], 2005) and sex is considered an important risk factor for cannabis use (Cooper and Craft, 2017). Among 15–16-years-old students, lifetime cannabis use is higher in males than in females and the male to female ratio (M/F) of lifetime cannabis use increases even further among all adults (M/F: 1.25–4.0) (European Monitoring Centre for Drugs and Drug Addiction [EMCDDA], 2005). Although males have a higher risk of developing CUD (Zhu and Wu, 2017), progression toward CUD is slightly faster in females than in males (Khan et al., 2013; European Monitoring Centre for Drugs and Drug Addiction [EMCDDA], 2017). Males also show different cannabis use patterns as compared to females and appear to use cannabis more frequently and at higher amounts (Cuttler et al., 2016). However, a faster progression to problematic cannabis use (Cooper and Haney, 2014) and more severe withdrawal symptoms (Levin et al., 2010) could explain why women typically show greater propensity to relapse to drug use than men (Becker and Hu, 2008; Fattore et al., 2008).

The fact that differences in cannabis use between males and females vary across countries suggests an influence of environmental (i.e., socio-cultural) factors. However, animal studies clearly indicate that biological factors, such as sex hormones and chromosomes, are significant modulators of drug sensitivity (Quinn et al., 2007; Marusich et al., 2015). In keeping with this, gender-tailored detoxification treatments and relapse prevention strategies for patients with CUD are increasingly requested (Fattore, 2013).

## Sex Steroid Hormones

Sex differences arise because of differences in sex chromosomes. The presence of the sex-determining region of Y (Sry) gene on the Y chromosome induces testicular development and consequently the production of testosterone (Polanco and Koopman, 2007). Testosterone and its derivative dihydrotestosterone (DHT) are responsible for the development of the male phenotype. Absence of the Sry gene leads to the development of ovaries that produce estradiol and progesterone. Estrogens, progesterone and testosterone have a strong impact on sexual differentiation, maturation and adult sexual behavior (Arnold and Breedlove, 1985; McEwen et al., 1987; Wallen, 1990; Meisel and Sachs, 1994; Hull et al., 1999; Morris et al., 2004; Becker, 2009; Argiolas and Melis, 2013; Motta-Mena and Puts, 2017). The presence of sex hormones during development gives rise to various organizational differences in the male and female brain, which ultimately affect reproductive and non-reproductive behavior (Beatty, 1979).

Sex hormones are synthesized by conversion of cholesterol into pregnenolone, which is the precursor of all steroid hormones (Hanukoglu, 1992). Interestingly, pregnenolone protects the brain from cannabinoid type-1 receptor (CB1R) overactivation, by acting as a potent endogenous allosteric inhibitor of CB1Rs (Vallée et al., 2014), and prevents cannabinoid-induced psychosis in mice (Busquets-Garcia et al., 2017). Sex hormones can be divided into three main subtypes with distinct molecular functions and sexually dimorphic expression and distribution: androgens (e.g., testosterone, dehydroepiandrosterone, androstenedione), estrogens (e.g., 17-alpha and 17-beta estradiol, estrone, estriol) and progestogens (e.g., progesterone, allopregnanolone, pregnenolone) (Figure 2). Sex hormones are produced by the gonads in response to the stimulating activity of the pituitary gonadotropins whose release is, in turn, under the control of the hypothalamic gonadotropin releasing hormone (GnRH). At the central level, several neurotransmitters are able to modify the release of GnRH, including norepinephrine, dopamine, serotonin, gamma-aminobutyric acid (GABA) and glutamate (Sagrillo et al., 1996). Cannabinoids were found to significantly modulate the activity of the hypothalamic-pituitary-gonadal (HPG) and -adrenal (HPA) axes (Brown and Dobs, 2002) and their interactions (Karamikheirabad et al., 2013). Interestingly, sex hormones influence the action of cannabinoids on these axes (López, 2010) suggesting bidirectional interactions between sex hormones and the endocannabinoid system (Table 1).

**Table 1:** Main findings from representative studies investigating the interaction between the endocannabinoid system and the sex or ASS hormones.

Main finding(s)	Reference
THC accumulates in testes in rats	Ho et al., 1970
Chronic consumption of cannabis significantly lowers plasma testosterone levels in humans	Kolodny et al., 1974
THC exerts its influence on rodent sexual behavior by exerting centrally mediated effects	Gordon et al., 1978
Acute administration of THC inhibits luteinizing hormone (LH) in males and females across a variety of mammalian species (from mice to monkeys)	Nir et al., 1973; Chakravarty et al., 1975; Ayalon et al., 1977; Besch et al., 1977; Chakravarty et al., 1982; Dalterio et al., 1983
Cannabinoids suppress GnRH secretion by modulating the activity of neurotransmitters involved in the regulation of GnRH secretion	Steger et al., 1983; Murphy et al., 1994
Brain CB1R expression significantly differs between males and females and displays a strong sex hormone-dependent modulation in female rats	Rodríguez de Fonseca et al., 1994
The content of the endocannabinoid AEA and 2-AG significantly differs between males and females and is affected by hormonal cycling in female rats	González et al., 2000; Bradshaw et al., 2006
Estrogen inhibits FAAH *in vitro* and *in vivo*	Maccarrone et al., 2000; Waleh et al., 2002
Sex hormones (progesterone), CB1Rs and D1Rs interact to regulate female rodents’ sexual behavior, and possibly, other motivated behaviors	Mani et al., 2001
AEA suppresses LH and testosterone levels in WT, but not CB1R-KO mice	Wenger et al., 2001
The inhibitory effects of cannabinoids on HPG axis function are reversed by estrogen	Scorticati et al., 2004
Immortalized GnRH neurons *in vitro* are capable of synthesizing endocannabinoids which exert immediate negative feedback control over GnRH secretion	Gammon et al., 2005
The anabolic steroid nandrolone blocks THC-induced CPP and increases the somatic manifestations of THC precipitated withdrawal	Célérier et al., 2006
The ovarian hormones significantly affect cannabinoid seeking and taking behavior in rats	Fattore et al., 2007, 2010
Systemic administration of the CB1R antagonist AM251 blocks the orexigenic effect of testosterone	Borgquist et al., 2015
Testosterone in adult males and estradiol in adult females modulate THC metabolism	Craft et al., 2017
Nandrolone modifies cannabinoid self-administration and brain CB1R density and function	Struik et al., 2017

*2-AG, 2-arachidonoylglycerol; AEA, anandamide; AAS, androgenic anabolic steroids; CB1R, cannabinoid sub-type 1 receptor; CPP, conditioned place preference; D1R, dopamine sub-type 1 receptor; FAAH, fatty acid amide hydrolase; GnRH, Gonadotropin Releasing Hormone; HPG, hypothalamic–pituitary–gonadal; KO, knock-out; LH, luteinizing hormone; WT, wild type.*

The main molecular targets of sex hormones are members of the nuclear hormone receptor family, which areligand-activated transcription factors involved in the regulation of gene expression (Mangelsdorf et al., 1995). Testosterone, estrogen and progesterone target the androgen receptors (ARα and ARβ), the estrogen receptors (ERα and ERβ) and the progesterone receptor, respectively, although considerable receptor “*promiscuity*” might exist in each case. Nuclear receptors are ubiquitously expressed in the central nervous system (CNS), including areas associated with reward and addiction (Bookout et al., 2006). Besides transcriptional effects, sex hormones are also reported to have fast non-genomic actions by modulating the activity of G protein-coupled receptors (GPRCs), ion channels and signaling proteins (Simoncini and Genazzani, 2003).

Sex hormones cause permanent organizational sex differences that are fixed during early development but they also maintain certain sex differences during the adult phase as long as these hormones are present, i.e., induce activational effects (McCarthy et al., 2012). For example, gonadectomy in adulthood completely suppresses sexual behavior in males and receptive and proceptive behaviors in females, all effects being reverted by exogenous hormonal replacement (Micheal and Wilson, 1974; Mitchell and Stewart, 1989; Jones et al., 2017). When released, sex hormones are also able to deeply influence the organization and activity of one of the most important target organs of hormonal action, which is the brain (Arnold and Breedlove, 1985; McEwen and Milner, 2017). Gonadal hormones thus provide a biological basis for sex differences in endocannabinoid-related behaviors and are expected to contribute to the sexual dimorphic actions of cannabinoids (Craft and Leitl, 2008; Craft et al., 2013).

## Sex Differences in the Endocannabinoid System

The endocannabinoid system is an evolutionary conserved signaling system that modulates multiple functions and consists of cannabinoid receptors, endogenous ligands (i.e., endocannabinoids) and several enzymes involved in the synthesis and degradation of endocannabinoids. The receptors and endogenous ligands of the endocannabinoid system were discovered in the late ‘80s and early ’90s, respectively. CB1Rs are highly expressed in the brain (Tsou et al., 1998; Freund et al., 2003; Howlett et al., 2004) and are considered the main type of receptor mediating cannabinoid signaling in response to exposure to THC (Moldrich and Wenger, 2000). CB1Rs are also highly expressed in fat tissue which might explain their role in energy homeostasis regulation, while cannabinoid type-2 receptors (CB2Rs) are predominantly expressed in cells of the immune system (van der Stelt and Di Marzo, 2005). CB1Rs and CB2Rs are GPCR and can be activated by THC or endogenous cannabinoid ligands like anandamide (AEA) and 2-arachidonylglycerol (2-AG) (McPartland et al., 2007). Activation of cannabinoid receptors results in the modulation of several signals, including inhibition of adenylate cyclase, activation of the MAPK pathway, stimulation of inwardly rectifying K^+^ channels, and inhibition of voltage-activated Ca^2+^ channels. Ultimately, cannabinoid receptor activation modulates the activity of most neurotransmitter systems, including GABA, glutamate, dopamine, and serotonin (van der Stelt and Di Marzo, 2003). The tonic 2-AG signaling at inhibitory inputs onto dopamine neurons has been shown to differ between sexes (Melis et al., 2013), supporting the notion that there are quantitative differences in the endocannabinoid system in males and females which likely contribute to altered cannabinoid sensitivity. Noteworthy, several sex differences in the endocannabinoid system are related to changes in steroid hormone levels and activity.

Sex hormones can affect the activity of several neurotransmitters in the CNS, including the endocannabinoid functioning (Nguyen et al., 2017; Moraga-Amaro et al., 2018), and significant sex-dependent differences in CB1R density and function have been described (reviewed in Antinori and Fattore, 2017). In a pioneering work, Rodríguez de Fonseca et al. (1994) investigated the expression of brain CB1Rs in male and female rats under different hormonal conditions and reported higher CB1R binding in males than females in almost all the brain areas investigated (i.e., striatum, limbic forebrain, and mesencephalon). Notably, CB1R binding in males was not affected by gonadectomy and/or testosterone replacement, while in females a strong sex hormone-dependent modulation of CB1R expression was observed, with ovariectomy increasing CB1R affinity in the striatum and decreasing CB1R density in the limbic forebrain (Rodríguez de Fonseca et al., 1994). González et al. (2000) found that males have higher levels of CB1R-mRNA transcripts than females in the anterior pituitary gland but that, in females, CB1R-mRNA transcripts fluctuate during the different phases of the ovarian cycle with the highest expression on the second day of diestrus and the lowest expression on estrus. Based on these findings it was suggested that higher levels of estrogen in the anterior pituitary gland could serve to inhibit CB1R expression, reducing the inhibitory endocannabinoid tone within the HPG axis around the time of ovulation (López, 2010). More recently, Castelli et al. (2014) found that CB1R density was significantly lower in the prefrontal cortex (PFC) and amygdala of cycling females compared to males and ovariectomized (OVX) females, and that administration of estradiol to OVX markedly reduced the density of CB1Rs to the levels observed in cycling females. In addition, OVX females displayed higher CB1R function in the cingulate cortex compared to intact and OVX + estradiol females. Interestingly, sex and estradiol also affected motor activity, social behavior and sensorimotor gating (Castelli et al., 2014), which are behaviors sensitive to the effects of different classes of drugs of abuse, in line with the idea that females can represent a more vulnerable phenotype (at neurochemical and behavioral level) than male rats in developing addiction-like behaviors. In addition, estradiol time-dependently modulates CB1R binding in brain structures that mediate nociception and locomotor activity (Wakley et al., 2014).

Besides impacting on CB1R expression, sex hormones regulate the levels of endocannabinoids. Bradshaw et al. (2006), for example, measured the levels of AEA and 2-AG in several brain areas (i.e., pituitary gland, hypothalamus, thalamus, striatum, midbrain, hippocampus, and cerebellum) in male rats and in females at five different time points along the estrous cycle. They found that AEA content was higher in females than males in both the anterior pituitary gland and hypothalamus (Bradshaw et al., 2006). With the exception of the cerebellum, all brain regions examined revealed significant differences along the estrous cycle in the level of at least one endocannabinoid, with changes occurring predominantly within the 36-h time period surrounding ovulation and behavioral estrus. In general, studies on the regulatory role of sex hormones on the endocannabinoid system failed to provide a clear and linear relationship between the two, and rather showed that these relations are quite complex and depend largely on the specific aspect considered (i.e., receptor affinity or density), the specific endocannabinoid (i.e., AEA or 2-AG) or the brain area investigated (Gorzalka et al., 2010; Gorzalka and Dang, 2012). The interpretation of these findings is further complicated by the fact that (i) all studies performed behavioral testing and/or tissue and serum collection at different time points after gonadectomy, (ii) animals were of different strains and tested at different ages (although they were adult in all studies), and that (iii) animals were kept under hormonal replacement regimen for different periods of time (1 day–3 weeks) before testing. However, the following findings are consistent among studies: (i) higher density of CB1Rs in male hypothalamus and limbic areas coupled, in general, with lower levels of endocannabinoids; (ii) there are significant differences along the hormonal cycle of females, with major changes occurring in the expression of CB1Rs in pituitary gland, hypothalamus and midbrain limbic structures when passing from diestrus to proestrus and behavioral estrus.

Sex steroids, like estrogens, can also regulate the activity of the endocannabinoid metabolizing enzymes. Fatty Acid Amide Hydrolase (FAAH) is the main enzyme involved in the degradation of AEA (Patel et al., 2017). The promoter region of the FAAH gene contains an estrogen binding response element, and translocation of the estrogen receptor to the nucleus results in repression of FAAH transcription *in vitro* (Waleh et al., 2002) and *in vivo* (Maccarrone et al., 2000). Ovariectomy prevents the estrogen-induced down-regulation of FAAH expression, and both progesterone and estrogen reduce basal levels of FAAH (Maccarrone et al., 2000). The impact of estrogen-mediated regulation of FAAH activity at behavioral and neurochemical level is still under investigation (Hill et al., 2007).

In humans, plasma AEA levels fluctuate across the menstrual cycle, with a peak at ovulation and the lowest plasma AEA levels observed during the late luteal phase (El-Talatini et al., 2010). In addition, significant positive correlations exist between plasma levels of AEA and plasma levels of estradiol, luteinizing (LH) and follicle-stimulating hormone (FSH) levels (El-Talatini et al., 2010). More recently, brain imaging studies revealed sex differences in the endocannabinoid system. By using positron emission tomography (PET) and the CB1R-selective radioligand [(11)C]OMAR it was shown that CB1R availability is higher in healthy females than in males (Neumeister et al., 2013; Normandin et al., 2015). In addition, it was reported that anandamide levels are lower in females than males (Neumeister et al., 2013). Another study combined PET with the CB1R-selective radioligand [18F]MK-9470 to examine CB1R binding in healthy men and women (van Laere et al., 2008). In this study, CB1R binding was higher in males than in females in all the brain areas investigated and strongly increased with aging in females, suggesting that age-dependent changes in the levels of sex hormones can control CB1R binding in females (van Laere et al., 2008).

While some of the sexual dimorphisms in the brain endocannabinoid system might be permanent, cannabinoid sensitivity is not fixed and can be acutely modulated by hormone-dependent fluctuations of CB1R density, levels of endocannabinoids and of endocannabinoid metabolizing enzymes. Collectively, the hormone-driven sexual dimorphic endocannabinoid system provides a biological basis for sex differences in endocannabinoid-related behaviors, including reward-related behavior (Fattore and Fratta, 2010; Fratta and Fattore, 2013).

## Sex Differences in Cannabinoid Action

Numerous studies show sex differences in functions in which the endocannabinoid system is involved, which span from regulation of motivated behaviors, like sex activity (Gorzalka et al., 2010; López, 2010; Androvicova et al., 2017) and food intake (Farhang et al., 2009), to locomotor and exploratory activity (Craft and Leitl, 2008; Craft et al., 2017), nociception (Tseng and Craft, 2001; Craft et al., 2017), working memory (Crane et al., 2013), anxiety (Viveros et al., 2011; Bowers and Ressler, 2016) and vulnerability to develop addictive disorders (Fattore et al., 2014; Marusich et al., 2014; Becker, 2016). Endocannabinoids are also directly involved in the anxiolytic effects of estrogen; in turn, estrogen may elicit changes in emotional behavior through an endocannabinoid mechanism (Hill et al., 2007).

Sexual maturation takes place under hormonal control during puberty and adolescence. Due to the deep changes occurring during these periods of life, individuals of both sexes are particularly (although differentially) sensitive to many stimuli, vulnerable toward the development of psychopathological conditions and more prone to abuse drugs, including cannabis (Wiley and Burston, 2014; Silva et al., 2016; Wagner, 2016). Exposure to cannabinoids during critical developmental periods alters several functions in adult animals (Schneider, 2008; Rubino and Parolaro, 2016), including working (Schneider and Koch, 2003; O’Shea et al., 2004) and spatial memory (Rubino et al., 2009), sensorimotor gating (Schneider and Koch, 2003), anxiety and anxiolytic-like responses (Biscaia et al., 2003; O’Shea et al., 2004; Viveros et al., 2005b), anhedonia, depressive-like states (Schneider and Koch, 2003; Rubino et al., 2008) and sexual behavior (Chadwick et al., 2011). Long-term alterations induced by cannabinoids in the developing organism are well known (Gupta and Elbracht, 1983; Navarro et al., 1994; Pistis et al., 2004; Viveros et al., 2005a; Spano et al., 2006; Ellgren et al., 2007; Renard et al., 2014; Rubino and Parolaro, 2016; Melas et al., 2018) and recent reports are pointing out epigenetic mechanisms underlying cannabis action (Szutorisz and Hurd, 2016, 2018; Prini et al., 2017). Yet, researchers started only recently to unravel sexually dimorphic long-term effects of early cannabinoid exposure on behavior, cognition and emotional states (Viveros et al., 2011; Lee et al., 2014; Keeley et al., 2015a,b).

For instance, the ability of sex hormones to affect cannabinoid self-administration was established only recently. Such a delay is probably due to the fact that human cannabis use is extremely difficult to model in laboratory animals (Panlilio et al., 2010) and that the development of reliable protocols of cannabinoid self-administration in mice (Martellotta et al., 1998), rats (Fattore et al., 2001) and monkeys (Justinova et al., 2003) has taken long time and efforts. Importantly, these models made it possible to investigate factors that modulate spontaneous cannabinoid intake in animals, including strain (Deiana et al., 2007) and sex (Fattore et al., 2007, 2010). Notably, female rats are able to discriminate THC from vehicle at a lower dose and faster rate than male rats (Wiley et al., 2017), although no significant sex differences were observed in the cannabinoid place preference test (Hempel et al., 2017). Moreover, ovarian hormones were identified as important modulators of cannabinoid self-administration, since bilateral ovariectomy significantly reduced drug-taking and drug-seeking in female rats (Fattore et al., 2007, 2010). Unfortunately, which specific sex hormone is able to finely modulate cannabinoid intake is still uncertain, highlighting the need for studies that combine gonadectomy with hormone replacement.

The effects of hormonal fluctuation during the menstrual cycle on the responses to drugs of abuse have been consistently investigated (Terner and de Wit, 2006; Carroll et al., 2015; Weinberger et al., 2015). Yet, the influence of sex hormones and menstrual cycle on the subjective and objective effects of marijuana has only been occasionally studied in female smokers. For example, Griffin et al. (1986) found no effect of the specific phase of the menstrual cycle on marijuana intake, a finding consistent with the negative results reported by Lex et al. (1984) which monitored cannabis-induced changes in pulse rate and mood in women during the follicular, ovulatory and luteal phases of the cycle. These earlier studies, however, failed to detect strong hormonal-dependent effects of marijuana intake along the menstrual cycle, and more controlled studies are needed before reaching any definitive conclusion on hormonal influences on cannabinoid use and sensitivity.

## (Endo)Cannabinoids, Sex Hormones and Dopamine

Sex hormones have been found to be important modulators of several drugs of abuse (Lynch et al., 2000; Carroll et al., 2004; Fattore et al., 2007, 2008; Lynch, 2008; Carroll and Lynch, 2016; Swalve et al., 2016). Estradiol and progesterone rapidly induce changes in dopaminergic signaling within the dorsal striatum and nucleus accumbens of female rats (Becker, 1999), effects that are important for the regulation of normal physiological states and relevant reproductive behaviors (Yoest et al., 2018). While the enhancing effect of ovarian hormones on drug craving has been traditionally attributed to estrogens (even in view of their ability to elicit direct dopamine release in the brain), it was suggested that progesterone, rather than estradiol, is responsible for the reducing effect on drug-seeking behavior (Feltenstein and See, 2007; Feltenstein et al., 2009; Carroll and Lynch, 2016).

As discussed above, brain CB1R distribution, synthesis of endogenous cannabinoids and activity of enzymes involved in cannabinoid metabolism (turnover) are significantly affected by sex hormones. At systems level, hormone-dependent differences and fluctuations in cannabinoid function may directly affect the activity of brain neurotransmitters and structures involved in cognitive and emotional aspects of motivated behaviors (Schultz, 1997; Berridge and Robinson, 1998; Ikemoto and Panksepp, 1999; Salamone and Correa, 2002; Goto and Grace, 2005; Cheng and Feenstra, 2006; Di Chiara and Bassareo, 2007; Berridge et al., 2009), like feeding (Melis et al., 2007; Bassareo et al., 2015; Fois et al., 2016; Coccurello and Maccarrone, 2018; Contini et al., 2018) and sexual behavior (Pfaus et al., 1990; Pfaus and Everitt, 1995; Sanna et al., 2015, 2017) as well as psychopathological states (Dunlop and Nemeroff, 2007; Maia and Frank, 2017) and addiction-like behaviors (Everitt and Robbins, 2005, 2016). Such a modulation can happen (i) by a direct interaction of the cannabinoid system with the mesolimbic dopaminergic system, the core component of the neurobiological substrates at the basis of motivated behavior (Gardner, 2005; Fadda et al., 2006; Lecca et al., 2006; Zangen et al., 2006; Melis and Pistis, 2007; Panagis et al., 2014; Bloomfield et al., 2016; Maldonado et al., 2006), or (ii) by indirect actions in limbic areas (e.g., hippocampus, amygdala, PFC) strictly interconnected with mesolimbic dopaminergic neurons through (mainly) glutamatergic projections to the ventral tegmental area and nucleus accumbens (Laviolette and Grace, 2006; Laviolette, 2017). Sex hormones can modulate cannabinoid influence on motivated behaviors and stress responses by acting also at the level of several hypothalamic nuclei (Cota, 2008).

The leading hypothesis that sex steroids and (endo)cannabinoid actions can converge on the dopaminergic mesolimbic system to regulate important motivational aspects in a sexually dimorphic manner deserves further confirmation. To date, it explains interactions of cannabinoids and sex hormones only at the level of specific brain systems, while most of the information at molecular and genetic/epigenetic level are still missing, although initial efforts in this direction have begun to fill the gap (see for example Mani et al., 2001; Gammon et al., 2005; Szutorisz and Hurd, 2016, 2018; Prini et al., 2017; Rosas et al., 2018). Furthermore, this hypothesis takes into account almost exclusively the cannabinoid effects mediated by central CB1Rs, but CB2Rs may also play a part through their actions on brain dopamine systems (Liu et al., 2017). The importance of sex hormones in modulating drug sensitivity is further supported by studies that have shown a clear association between exposure to synthetic male steroids and drug sensitivity.

## Anabolic-Androgenic Steroids and Cannabinoid Action

Anabolic-androgenic steroids are synthetic derivatives of the male hormone testosterone and are used therapeutically for the treatment of various diseases including hypogonadism, angioedema, anemia, osteoporosis, and muscle wasting (Basaria et al., 2001). Non-medical use of AAS is often observed among professional and non-professional athletes in order to improve physical appearance and enhance performance (Sagoe et al., 2014). Global lifetime prevalence rate of non-medical AAS use is estimated to be 3.3% (Sagoe et al., 2014). AAS doses used for non-medical purposes are typically much higher (10–100×) than doses for medical use and are associated with several physical and psychological side effects (Hartgens and Kuipers, 2004). Physical side effects that have been observed after use of AAS include infertility, baldness, breast development, severe acne, high blood pressure, blood clots, heart attack, and stroke (Hartgens and Kuipers, 2004). Possible psychological consequences of AAS use are increased aggression, anxiety, and depression (Hartgens and Kuipers, 2004). Clinical and epidemiological data show that AAS are often co-abused with addictive substances, including cannabis (DuRant et al., 1995; Arvary and Pope, 2000; Kanayama et al., 2003). Several reasons might explain why polypharmacy occurs in more than 95% AAS users (Parkinson and Evans, 2006). First, AAS users are known to take other drugs to counteract adverse side but they might also have a higher sensitivity toward substance abuse. Alternatively, AAS might have direct effects on various components of the brain reward system which alters the sensitivity of users toward other drugs of abuse.

Use of high doses of AAS can lead to addiction, which makes it conceivable that AAS are able to modulate the brain reward system (Kanayama et al., 2009). Although part of the rewarding effects of AAS might be derived from their effects on physical appearance, animal studies have shown that testosterone and other AAS can induce conditioned place preference in a dopamine receptor-dependent manner (Arnedo et al., 2000; Schroeder and Packard, 2000; Parrilla-Carrero et al., 2009) and increase self-administration behavior (Clark et al., 1996; Wood, 2004). In addition to their effects on reward-related behavior, AAS cause molecular and neurochemical changes in the dopaminergic, serotonergic and opioid neurotransmitter systems (Johansson et al., 1997; Kindlundh et al., 2001; Zotti et al., 2014) and alter the behavioral effects of different types of drugs (Kurling, 2008; Kurling-Kailanto, 2010; Kailanto, 2011).

Studies investigating the effects of AAS exposure on cannabinoid sensitivity are scarce at present. It was shown that testosterone significantly reduces THC-induced locomotor suppression or catalepsy in gonadectomized males (Craft and Leitl, 2008; Craft et al., 2017) and that chronic exposure to nandrolone, a derivative of testosterone also known as 19-nortestosterone (Figure 2), blocked THC-induced conditioned place preference in rats (Célérier et al., 2006). Further, we recently reported that chronic treatment of rats with nandrolone does not alter CB1R levels or function in several reward-related brain areas. However, when chronic nandrolone treatment is followed by cannabinoid self-administration, we observed a strong decrease in CB1R function in the hippocampus and a significant increase in cannabinoid intake (Struik et al., 2017). Given the profound effects that AAS have on various aspects of the molecular machinery of the brain reward system, it might come as no surprise that AAS also interfere with the rewarding properties of drugs of abuse, including cannabinoids.

Altogether, studies available so far suggest that AAS can have a repressing effect on the brain reward system, a notion that is strengthened by the observation that AAS reduce drug-induced neurochemical and behavioral effects of amphetamine, MDMA, THC, and cocaine, and increase voluntary alcohol and cannabinoid drug intake (Mhillaj et al., 2015). The hypothesized AAS-induced suppression of the reward system might result in the use of higher doses of drugs, which is associated with a higher addiction risk. It would be intriguing to find out to what extent blockade of steroid hormone activity contributes to prevent the repressive effect of these hormones in the reward system. Further studies are needed also to assess whether or not AAS can act as gateway drugs and lead to CUD and to better understand how they can impinge upon the endocannabinoid signaling within the brain.

## Conclusion

Cannabis is the most commonly used illicit drug worldwide and its use is associated with multiple adverse health effect including the risk of addiction. Identifying factors involved in cannabis use and abuse is critical for optimizing evidence-based prevention and treatment protocols. Similarly to other drugs of abuse, the prevalence of cannabis use and addiction differs between males and females, suggesting that sex is an important modulator of cannabinoid sensitivity. Accumulating evidence shows that the endocannabinoid system is sexually dimorphic and that sex hormones play a key role. Hormone-driven differentiation of the endocannabinoid system seems to provide a biological basis for sex differences in endocannabinoid-related behaviors, including reward-related behaviors. While sex differences in cannabinoid action are being increasingly studied in animals, controlled human studies are still limited. The endocannabinoid system is, for its intrinsic characteristics, a privileged target of the actions of both sex and anabolic-androgenic steroid hormones at different levels and, in turn, it can modulate the activity of sex hormones (Table 1). The observation that exposure to AAS causes dysfunction of the brain reward pathway in rats points to a potential risk factor for initiation of cannabis use, maintenance of regular use and development of CUD. The cross talk between endocannabinoid signaling and steroid hormones can occur differently in males and females, and many questions about underlying mechanisms remain unanswered, demanding further research in the field in an attempt to elucidate the basis of the sex differences often observed in cannabinoid sensitivity.

## Author Contributions

DS has developed the original idea and wrote the Introduction and the parts related to the risk factors for cannabis use and anabolic-androgenic steroids. FS wrote the parts related to sexual behavior, gonadal hormones, and dopamine-cannabinoid interactions. LF has developed the original idea, wrote the parts related to brain sexual dimorphisms and sex/gender differences and coordinated the work structuring of the different parts. All authors have approved the final version of the review.

## Conflict of Interest Statement

The authors declare that the research was conducted in the absence of any commercial or financial relationships that could be construed as a potential conflict of interest.
